# Supplementary material for: A synaptic mechanism for encoding the learned value of action-derived safety
Source: Nat Commun. 2026 Jun 4;17:4916. doi: 10.1038/s41467-026-73906-3 (PMC13237380; doi:10.1038/s41467-026-73906-3)
Supplement: Supplementary file 1 — Supplementary Information [file 41467_2026_73906_MOESM1_ESM.pdf]

SUPPLEMENTARY FIGURES

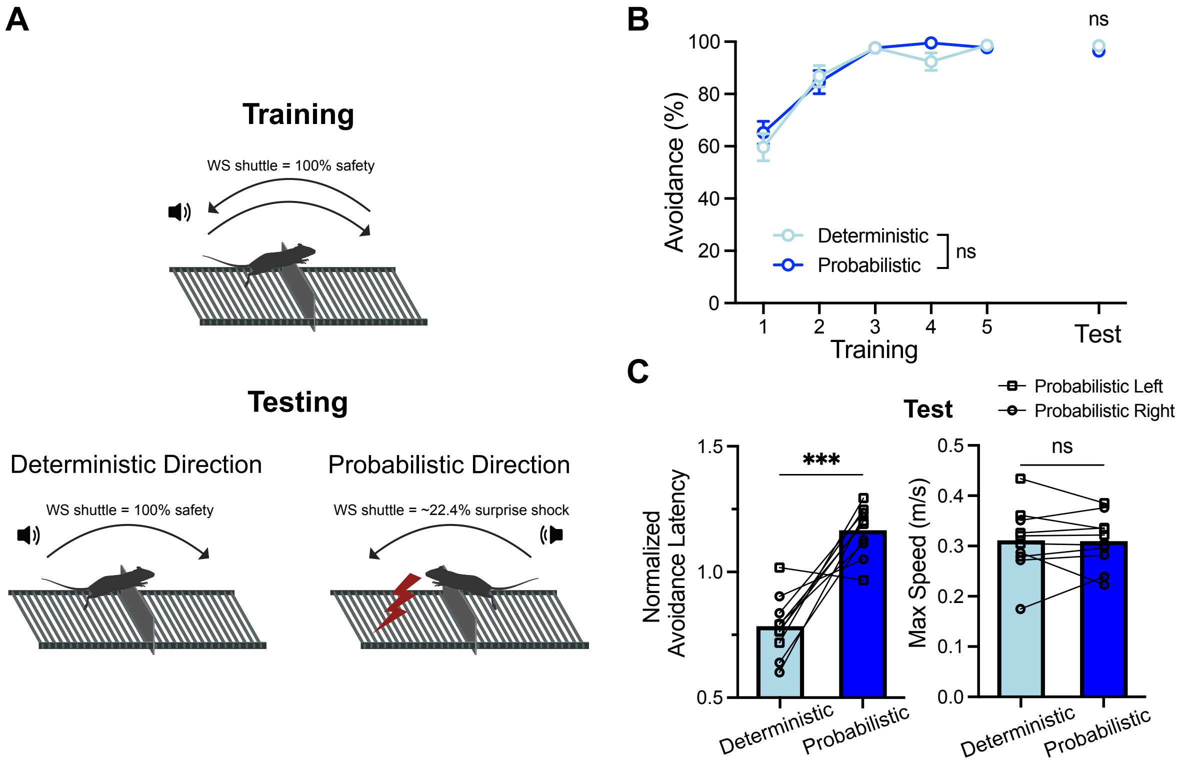

Supplementary Figure 1: Probabilistic safety decreases avoidance latency. (A) Schematic of training and testing protocol. Subjects were counterbalanced to side of surprise shock. **(B)** Avoidance rate across training and testing by direction. Training: 2-way Anova direction effect: p=0.0682, Testing: Paired t test: p=0.0657 (n=10 mice). **(C)** Avoidance latency on test day normalized to baseline trials preceding first surprise shock (left, Paired t test: p= 0.0002). Max speed of avoidance shuttle on test day (right, Paired t test: p= 0.9012). Data are shown as mean ± s.e.m. *p < 0.05, ***p < 0.001, ns: p > 0.05. Schematics created in BioRender. Macdonald, E. (2026) https://BioRender.com/4pm04zs. Source data are provided as a Source Data file.

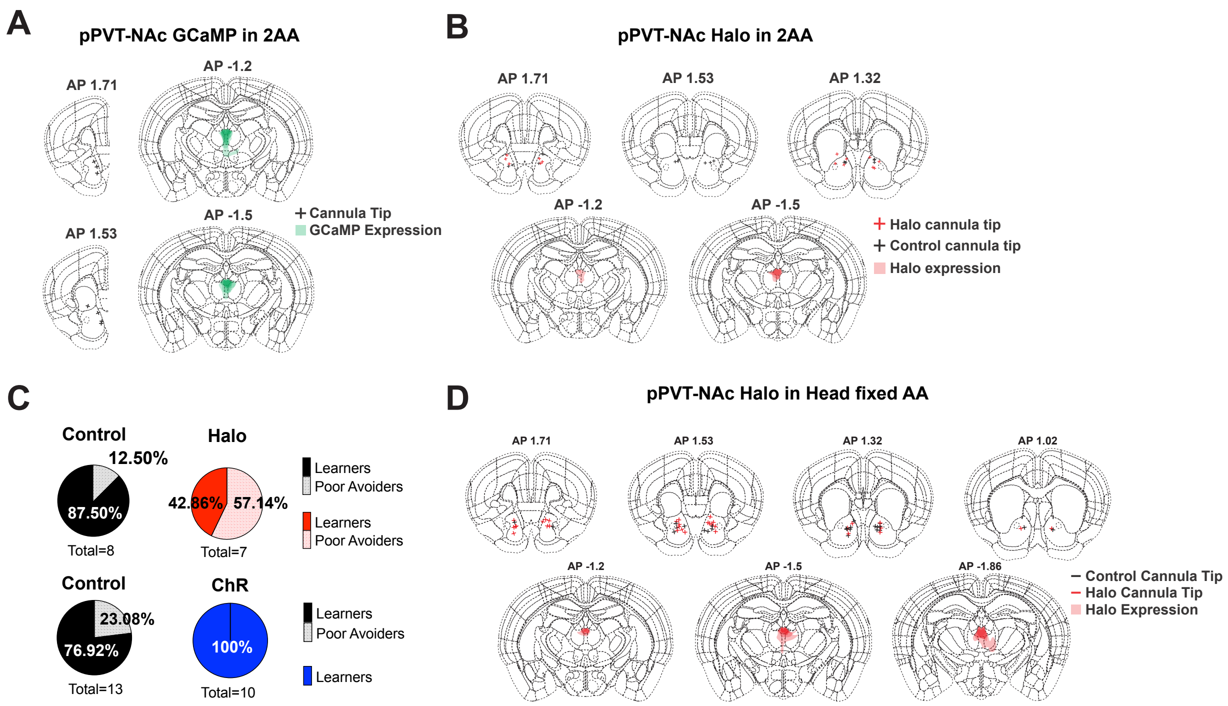

Supplementary Figure 2: Related to Figure 1. (A) Map of optic fiber cannula tip locations for photometry experiment in figures 1A-G (left). Map of viral spread of GCaMP at bregma -1.2 and -1.5 (right). **(B)** Map of optic fiber cannula tip locations for optogenetics experiment in figures 1H-L (top). Map of viral spread of halorhodopsin (Halo) at bregma -1.2 and -1.5 (bottom). **(C)** Proportion of poor avoiders (<30% avoidance day 3 of training) per optogenetic experimental group in figures 1H-L and S3. **(D)** Map of optic fiber cannula tip locations for optogenetics experiment in figures 1M-O (top). Map of viral spread of halorhodopsin at bregma -1.2 and -1.5 (bottom). Histological maps were adapted from the Allen Reference Atlas.

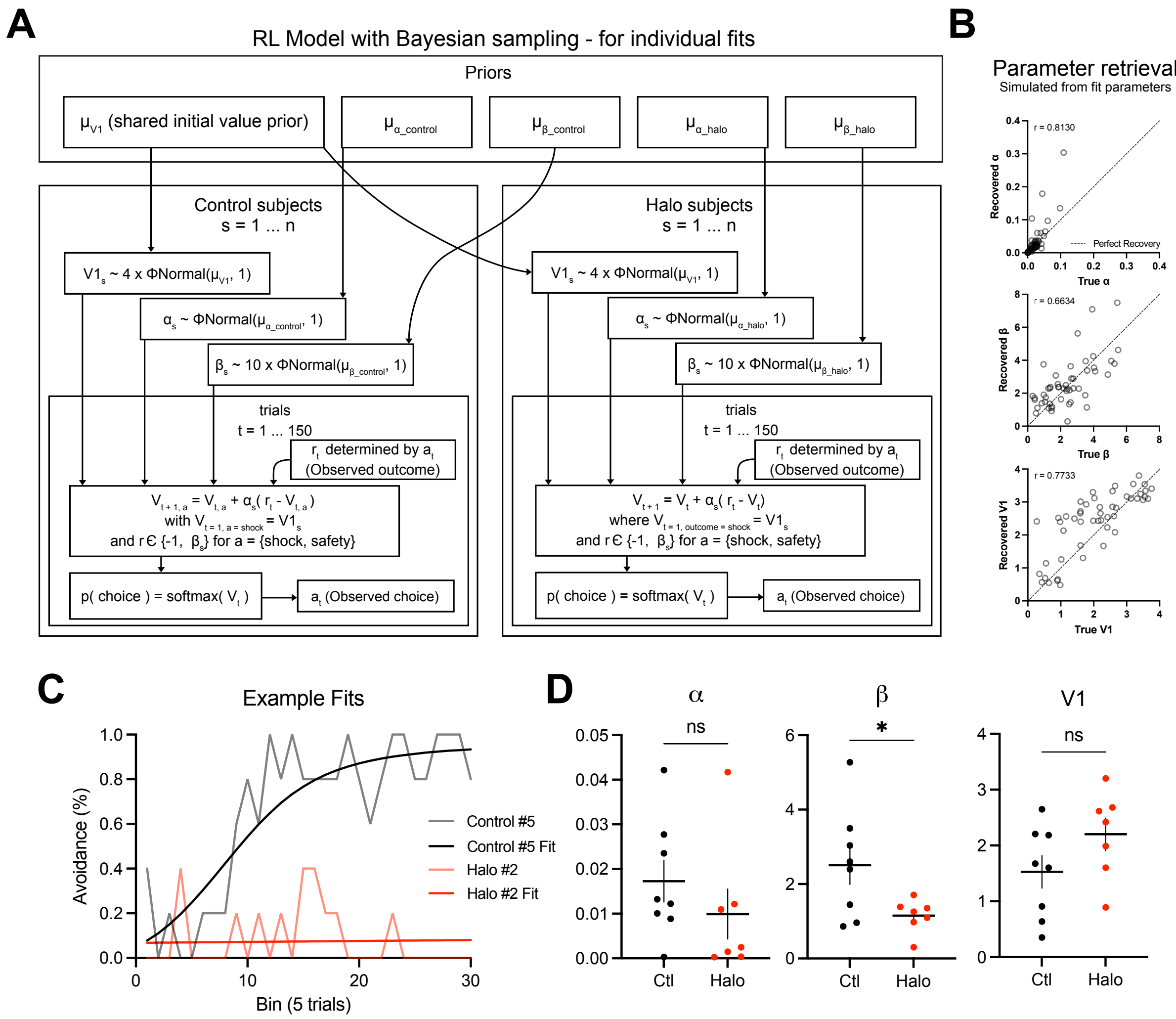

Supplementary Figure 3: PVT→NAc inhibition impairs safety value. (A) Schematic representing the hierarchical fit of a reinforcement model with Bayesian sampling. **(B)** Parameter retrieval performed for RL model to individuals. Starting parameters from fits to 50 mice run in 2AA across experiments. Pearson correlations: α $p<0.0001$; β $p<0.0001$; V1 $p<0.0001$. **(C)** Avoidance across 5 trial bins for representative control and Halo subjects and fit by Rescorla-Wagner model with Bayesian sampling. **(D)** Best fit parameters by subject. Unpaired t tests: α $p=0.1893$; β $p=0.0361$; V1 $p=0.1273$. *$p < 0.05$, ns: $p > 0.05$.

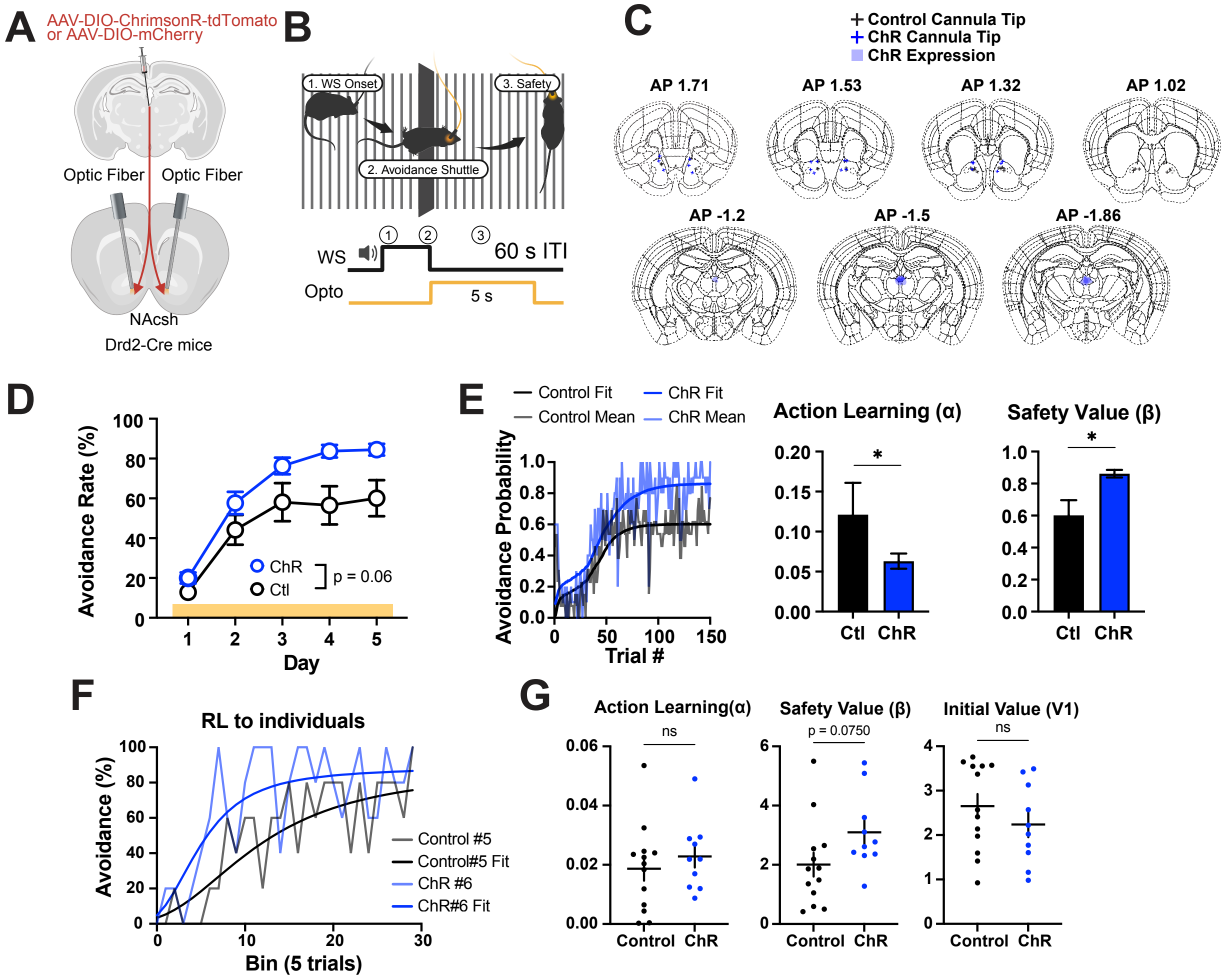
A
AAV-DIO-ChrimsonR-tdTomato
or AAV-DIO-mCherry
Optic Fiber
Optic Fiber
NAcsh
Drd2-Cre mice
B
1. WS Onset
2. Avoidance Shuttle
3. Safety
WS
Opto
60 s ITI
5 s
C
Control Cannula Tip
ChR Cannula Tip
ChR Expression
AP 1.71
AP 1.53
AP 1.32
AP 1.02
AP -1.2
AP -1.5
AP -1.86
D
Avoidance Rate (%)
ChR
Ctl
p = 0.06
Day
E
Control Fit
ChR Fit
Control Mean
ChR Mean
Avoidance Probability
Trial #
Action Learning (α)
Safety Value (β)
Ctl
ChR
F
RL to individuals
Avoidance (%)
Control #5
Control#5 Fit
ChR #6
ChR#6 Fit
Bin (5 trials)
G
Action Learning(α)
Safety Value (β)
Initial Value (V1)
ns
p = 0.0750
Control
ChR

Supplementary Figure 4: PVT→NAc activation boosts safety value. (A) Drd2-Cre mice were injected with ChrimsonR (ChR) or fluorescent control in PVT to bilaterally stimulate terminals in NAc. **(B)** Schematic of closed-loop optogenetic manipulation in the 2AA task (see Methods). **(C)** Map of optic fiber cannula tip locations (top). Map of viral spread of ChrimsonR at bregma -1.2, -1.5, and -1.86 (bottom). **(D)** Performance in the 2AA task with excitation of PVT-NAc at safety (Control n=13, ChR n=10 mice). 2-way Anova group effect: p=0.0554. **(E)** Avoidance probability across trial with best fit Rescorla-Wagner model probability by group (left). Best fit learning and value parameters by group with bootstrap estimated error (middle, right). Bootstrap permutation test for group difference: Learning p=0.0412; Value p=0.0208. **(F)** Avoidance across 5 trial bins for representative control and ChR subjects and fit by Rescorla-Wagner model with Bayesian sampling. **(G)** Best fit parameters by subject. Unpaired t tests: α p=0.4751; β p=0.075; V1 p=0.3100. Data are shown as mean ± s.e.m. *p < 0.05, ns: p > 0.05. Schematics created in BioRender. Macdonald, E. (2026) https://BioRender.com/4pm04zs. Histological maps adapted from the Allen Reference Atlas. Source data are provided as a Source Data file.

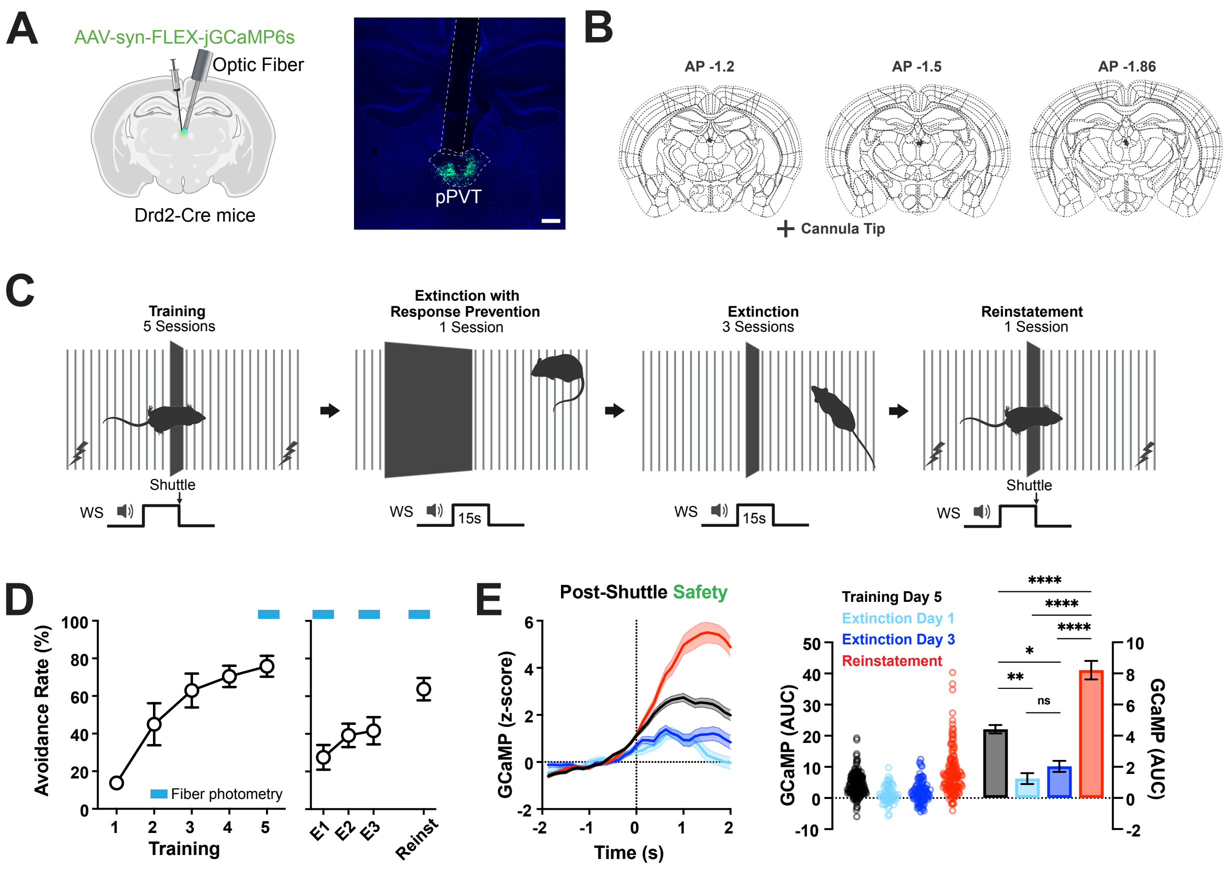

Supplementary Figure 5: PVT tracks safety devaluation. **(A)** Drd2-Cre mice were injected with GCaMP to record during 2AA and extinction (left). Representative image of injection and recording site (right). Scale bar, 200 µm. **(B)** Map of optic fiber cannula tip locations. **(C)** Schematic of training protocol (see Methods). **(D)** Performance in the 2AA task during training days 1-5, extinction (E1-3), and reinstatement (Reinst) (n=8 mice). **(E)** GCaMP dF/F z-score normalized at shuttle (safety) with optogenetic inhibition by day of training (left). Quantification of post event activity (right) with individual trials on the left y-axis and mean ± s.e.m. on the right y-axis. AUC, pairwise comparisons between groups, linear mixed-effects model for repeated measures: see statistics in Supp. Data 1 (n=495 trials from 8 mice). Data are shown as mean ± s.e.m. *$p < 0.05$, **$p < 0.01$, ****$p < 0.0001$, ns: $p > 0.05$. Schematics created in BioRender. Macdonald, E. (2026) https://BioRender.com/4pm04zs. Histological maps adapted from the Allen Reference Atlas. Source data are provided as a Source Data file.

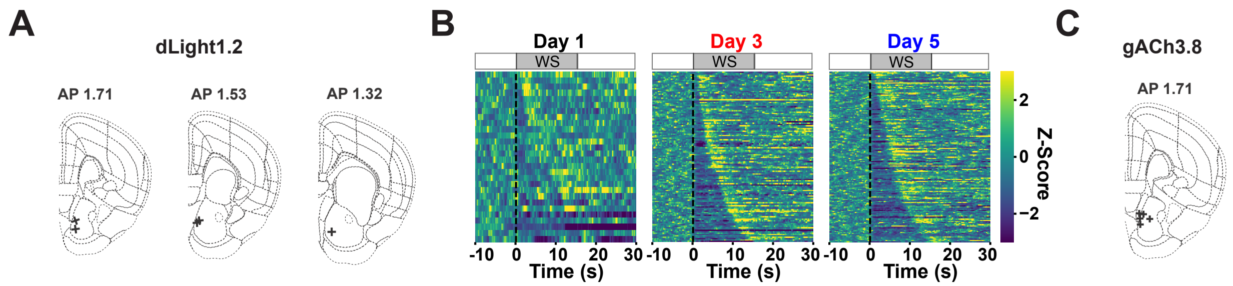

Supplementary Figure 6: Related to figures 2 and 3. (A) Map of fiber optic cannula tip location for dLight photometry experiment in figures 2A-F. **(B)** Heatmaps of dLight signals during avoidance trials, ordered by avoidance latency (low to high) for Day 1 (28 trials), Day 3 (124 trials), and Day 5 (154 trials). Related to figures 2A-F. **(C)** Map of fiber optic cannula tip location for gACh photometry experiment in figures 3D-J. Histological maps adapted from the Allen Reference Atlas.

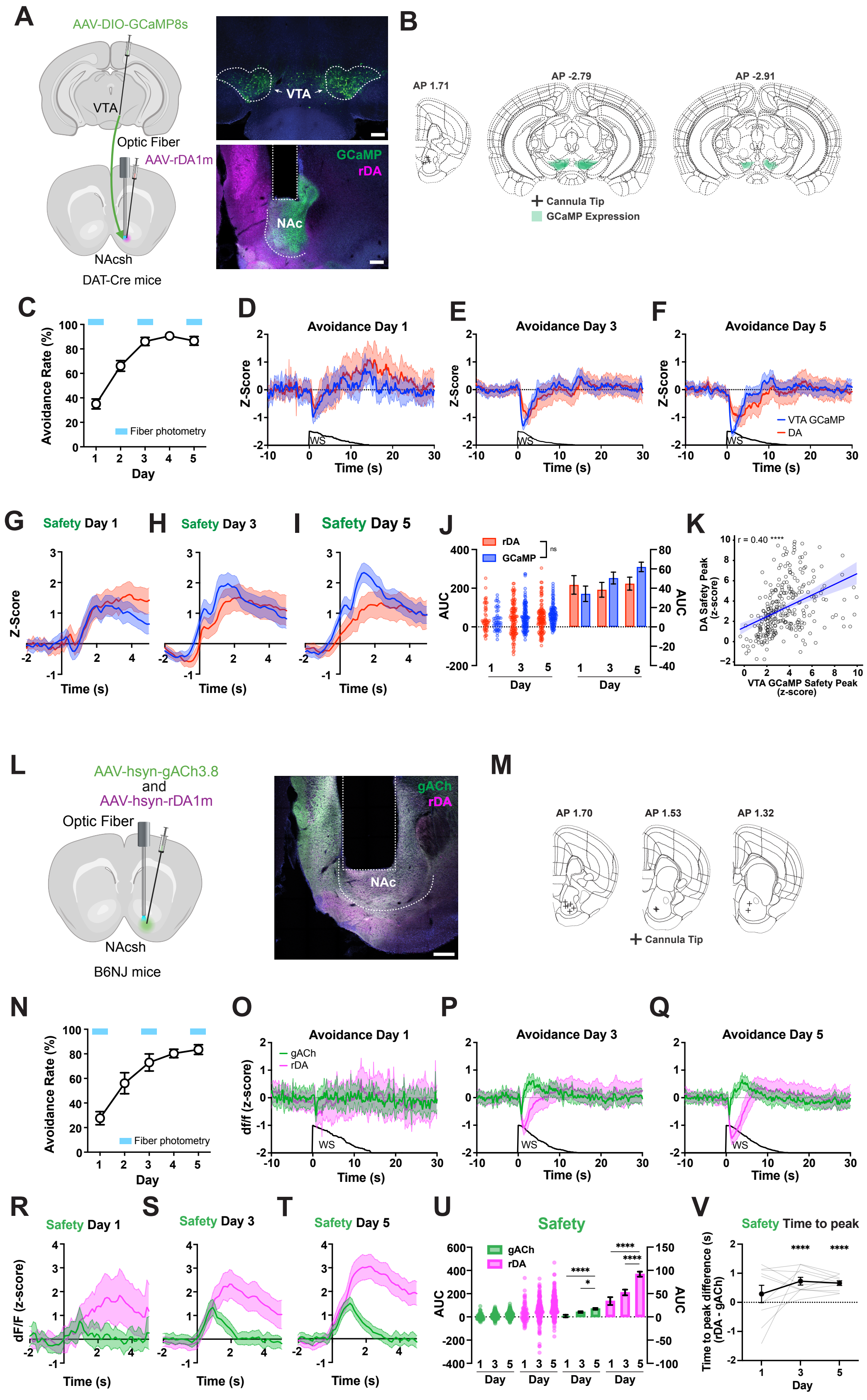
A
AAV-DIO-GCaMP8s
VTA
Optic Fiber
AAV-rDA1m
NAcsh
DAT-Cre mice
GCaMP
rDA
NAc
B
AP 1.71
AP -2.79
AP -2.91
Cannula Tip
GCaMP Expression
C
Avoidance Rate (%)
Fiber photometry
Day
D
Avoidance Day 1
E
Avoidance Day 3
F
Avoidance Day 5
Z-Score
Time (s)
WS
VTA GCaMP
DA
G
Safety Day 1
H
Safety Day 3
I
Safety Day 5
J
rDA
GCaMP
ns
AUC
K
r = 0.40 ****
DA Safety Peak (z-score)
VTA GCaMP Safety Peak (z-score)
L
AAV-hsyn-gACh3.8
and
AAV-hsyn-rDA1m
Optic Fiber
NAcsh
B6NJ mice
gACh
rDA
NAc
M
AP 1.70
AP 1.53
AP 1.32
Cannula Tip
N
O
P
Q
df/f (z-score)
gACh
rDA
R
S
T
dF/F (z-score)
U
Safety
V
Safety Time to peak
Time to peak difference (s) (rDA - gACh)

Supplementary Figure 7: rDA1m sensor activity matches dopaminergic terminals and slower time to peak than gACh at safety. (A) DAT-Cre mice were injected with GCaMP8s in the VTA and rDA1m in the NAc to validate the GRAB DA sensor (left). Representative images of injection and recording sites (right). Scale bars, 200 µm. **(B)** Map of optic fiber cannula locations (left) and GCaMP viral expression in VTA at bregma -2.79 and -2.91 (right). **(C)** Performance in the 2AA task (n=6 mice). **(D-F)** GCaMP (blue) and rDA (red) dF/F z-score normalized in all avoidance trials on training days 1 (**D**), 3 (**E**) and 5 (**F**), with average WS duration below. **(G-I)** dF/F z-score normalized at safety on training days 1 (**G**), 3 (**H**) and 5 (**I**). **(J)** Area under the curve quantification of 5s of safety post WS offset with individual trials on the left y-axis and mean ± s.e.m. on the right y-axis. AUC, pairwise comparisons, mixed effects model: see statistics in Supp. Data 1 (n=561 trials from 6 mice). **(K)** Correlation between peak z-score value at safety by trial of GCaMP and rDA. Pearson correlation: $p<0.0001$ (n=655 trials from 6 mice). **(L)** B6NJ mice were injected with 1:1 gACh3.8 and rDA1m in NAc to investigate neurotransmitter release dynamics (left). Representative image of recording site (right). Scale bar, 200 µm. **(M)** Map of optic fiber cannula locations. **(N)** Performance in 2AA task (n=10 mice). **(O-Q)** gACh (green) and rDA (magenta) dF/F z-score normalized in all avoidance trials on training days 1 (**O**), 3 (**P**) and 5 (**Q**), with average WS duration below. **(R-T)** dF/F z-score normalized at safety on training days 1 (**R**), 3 (**S**) and 5 (**T**). **(U)** Area under the curve quantification 5s of safety with individual trials on the left y-axis and mean ± s.e.m. on the right y-axis. AUC, pairwise comparisons, mixed effects model: see statistics in Supp. Data 1 (n=539 trials from 10 mice). **(V)** Time to maximum dF/F difference (rDA – gACh) during safety. Mixed effects model, intercept by day: see statistics in Supp. Data 1 (n=539 trials from 10 mice). Data are shown as mean ± s.e.m. Schematics created in BioRender. Macdonald, E. (2026) https://BioRender.com/4pm04zs. Histological maps adapted from the Allen Reference Atlas. Source data are provided as a Source Data file.

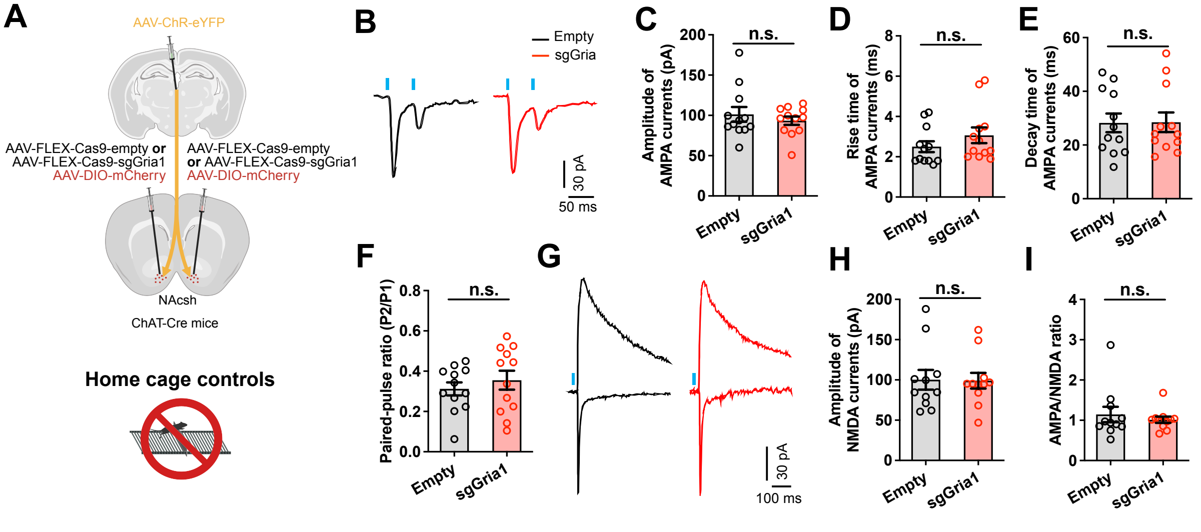

Supplementary Figure 8: NAc CIN Gria1 knockout does not change PVT-NAcCIN synapses before training. (A) ChAT-Cre mice were injected with channelrhodopsin in PVT, and FLEX-Cas9-sgGria1 or empty control bilaterally in NAc to record post-synaptic currents in CINs with PVT stimulation in a naïve condition. **(B)** Representative oEPSCs from control and knockout subjects. **(C-E)** Measurements of current properties. Mann-Whitney test: oEPSC amplitude p= 0.9323; Rise time p= 0.1721; Decay time p= 0.8874; Unpaired t-test: Paired-pulse ratio p= 0.2358. **(G)** Representative traces of AMPA and NMDA responses to PVT optogenetic stimulation. **(H-I)** Current amplitude measurements. Mann-Whitney test: NMDA amplitude p= 0.9353; AMPA/NMDA ratio p= 0.6994 (sgGria1 n=11-12 neurons from 2 mice, empty n=11-12 neurons from 2 mice). Data are shown as mean ± s.e.m. ns: p > 0.05. Schematics created in BioRender. Macdonald, E. (2026) https://BioRender.com/4pm04zs. Source data are provided as a Source Data file.

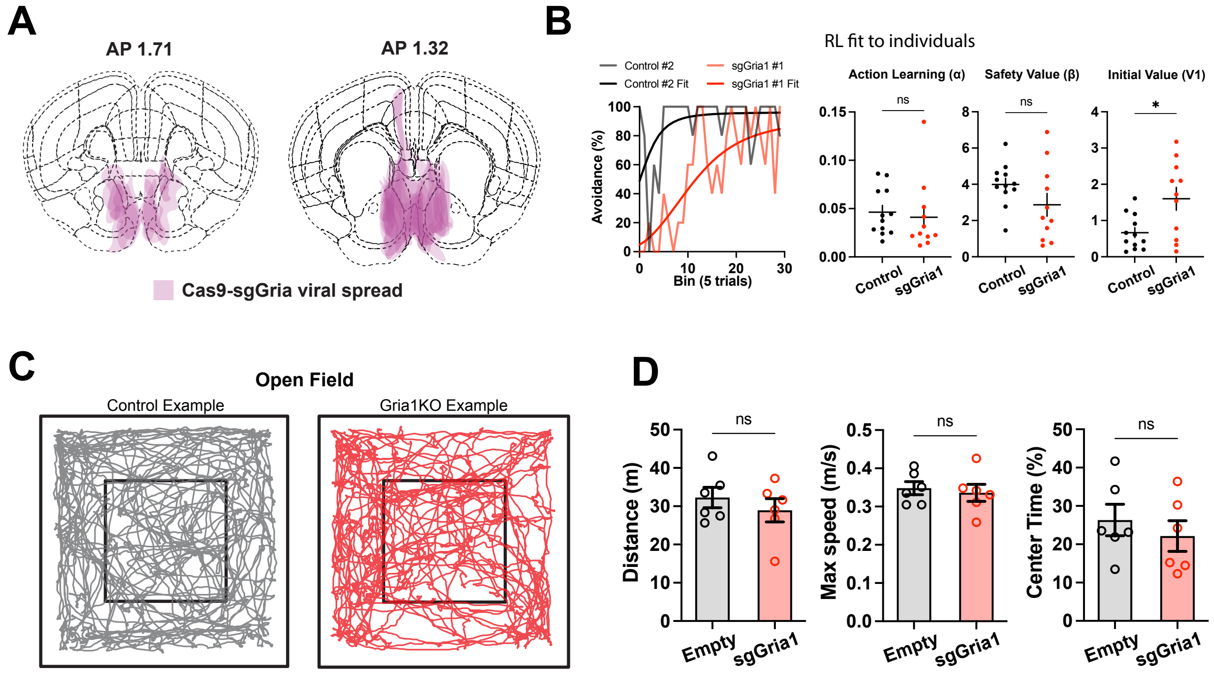

Supplementary Figure 9: Related to figure 5. (A) Map of viral spread for sgGria KO in figures 5N-Q at bregma 1.71 and 1.32. **(B)** Avoidance across 5 trial bins for representative control and sgGria1 subjects and fit by Rescorla-Wagner model with Bayesian sampling (left). Best fit parameters by subject. Mann Whitney test α p= 0.2351; Unpaired t tests: β p= 0.1226; V1 p= 0.0106. **(C)** Representative track paths for control (left) and sgGria1 (right) subjects in open field test for 10 minutes. **(D)** Locomotor or exploratory behavior unchanged by Gria1 knockout. Unpaired t tests: Distance p=0.4292; Max speed p=0.6775; Center time p=0.4811 (Control n=6, sgGria1 n=6 mice). Data are shown as mean ± s.e.m. *p < 0.05, ns: p > 0.05. Histological maps adapted from the Allen Reference Atlas. Source data are provided as a Source Data file.
