# Supplementary material for: A synaptic mechanism for encoding the learned value of action-derived safety
Source: Nat Commun. 2026 Jun 4;17:4916. doi: 10.1038/s41467-026-73906-3 (PMC13237380; doi:10.1038/s41467-026-73906-3)
Supplement: Supplementary file 2 — Description of Additional Supplementary Files [file 41467_2026_73906_MOESM2_ESM.pdf]

Description of Additional Supplementary Files

Supplementary Data 1 | Statistics table for all data presented in the manuscript
